# Radiographic artifacts in the diagnosis of dental caries: systematic review with meta-analysis

**DOI:** 10.1007/s11282-025-00879-2

**Published:** 2025-12-02

**Authors:** Mario Dioguardi, Ciro Guerra, Diego Sovereto, Angelo Martella, Khrystyna Zhurakivska, Angela Tisci, Fariba Esperouz, Maria Eleonora Bizzoca, Lorenzo Sanesi, Filiberto Mastrangelo, Lorenzo Lo Muzio, Nicola Cirillo, Domenico Ciavarella, Andrea Ballini

**Affiliations:** 1 Department of Clinical and Experimental Medicine, University of Foggia, Via Rovelli 50, Foggia, 71122 Italy; 2 DataLab, Department of Engineering for Innovation, University of Salento, Lecce, Italy; 3 Department of Life Science, Health and Health Professions, Link Campus University, Rome, Italy

**Keywords:** Artifacts analysis, Dental, Cervical burnout, Mach band effect, Periapical X-Rays, Caries

## Abstract

**Objectives:**

The radiographic diagnosis of dental caries is often complicated by the presence of radiographic optical effects, such as the Mach band effect and triangular-shaped radiolucencies (TSR). These phenomena can give rise to false-positive diagnoses, especially in bite-wing radiographs, thus influencing clinical decision-making and possibly leading to overdiagnosis and overtreatment. The aim of this systematic review and meta-analysis was to clarify the real prevalence of these radiographic optical effects in dental radiographs and to quantify their influence on diagnostic errors in caries detection.

**Methods:**

This systematic literature review follow the PRISMA guidelines and registered in PROSPERO (CRD420251083823), prior to its execution. Electronic searches were conducted on PubMed, Scopus and the Cochrane Library, with the addition of Grey literature and references of the main reviews on the topic. Inclusion criteria included clinical, in vitro and ex vivo studies evaluating the impact of TSR and Mach band effects on intraoral radiographs (bitewing or periapical), reporting diagnostic performance, prevalence or misinterpretation rates. Data extraction and risk of bias assessment (AXIS, QUADAS-2) were performed independently by two reviewers. Meta-analyses were performed using random effects models.

**Results:**

Of 640 identified reports, only five studies were included. The overall prevalence of non-carious TSR on maxillary molars was 26.44%, 270/1021. The meta-analysis showed that effects (TSR or Mach band) led to false positive diagnoses of caries or fractures, in approximately 13% of observations (60/464). Heterogeneity was high (I² > 90%) and the certainty of the evidence was classified as low to very low due to the type of studies included and the consistency indices.

**Conclusions:**

Radiographic optical effects, especially TSR and Mach band effect, are highly prevalent in bitewing images and significantly increase the risk of false positive caries diagnoses, especially in children. Given the low certainty of the available evidence, clinicians should interpret radiographic findings with caution and always correlate them with a thorough clinical examination. Further high-quality research is needed to develop standardized diagnostic criteria and strategies to mitigate radiographic optical effects in dental practice.

**Supplementary Information:**

The online version contains supplementary material available at 10.1007/s11282-025-00879-2.

## Introduction

The two main radiographic optical effects/phenomena that can lead to errors in the interpretation of intraoral radiographs, particularly in the diagnosis of dental caries, resulting in an overestimation of carious lesions, cervical burnout and the Mach band effect [1]. Cervical burnout is a radiographic effect that can mimic a carious lesion near the cementum-enamel junction (CEJ). It occurs when the X-ray beam passes tangentially across the curved proximal surface of the tooth, encountering less dental structure compared to other areas, thereby resulting in a localized apparent radiolucency [2]. This effect commonly appears between the CEJ and the alveolar crest, where the dentin is thinner, leading to reduced absorption of X-rays. The resulting image may therefore erroneously suggest the presence of a carious lesion. Besides, cervical burnout can be more pronounced in multirooted teeth, especially when the buccal and lingual roots do not perfectly overlap in the mesiodistal projection. A shallow root bifurcation may further accentuate this effect [3].

The Mach band effect, on the other hand, is an optical illusion that can complicate the radiographic diagnosis of caries. It arises when areas of differing density or brightness are adjacent, causing the human eye to perceive an exaggerated contrast at their interface. At the enamel-dentine junction (EDJ) on a bitewing radiograph, this may appear as an area of apparent radiolucency suggestive of caries. To reduce the risk of misinterpretation due to the Mach band, the operator may visually mask the more radiopaque enamel. If the band disappears following this masking, it is likely to represent a false-positive optical effect and, if it persists, may indicate a true lesion in the outer dentine [4].

However, the Mach-band effect is also involved in a characteristic radiographic finding termed non-carious triangular-shaped radiolucencies (TSR), which predominantly affect the mesial surfaces of maxillary molars (both deciduous and permanent), giving rise to diagnostic errors by being commonly mistaken for carious lesions [5].

The aim of this review is to systematically evaluate and elucidate the real impact of the Mach band effect and TSR on the radiographic diagnosis of dental caries. By critically appraising the available evidence, this systematic review seeks to clarify the extent to which these radiographic optical effects/phenomena may contribute to diagnostic inaccuracies, such as false-positive or false-negative findings, and to outline the strategies that can be employed to minimise their influence in clinical practice. Furthermore, the meta-analysis aims to highlight potential gaps in the current literature and propose recommendations for future research, with the ultimate goal of enhancing the accuracy and reliability of caries detection using intraoral radiography.

The Outcome is to determine the true impact of these two interrelated phenomena, the Mach-band effect and non-carious triangular-shaped radiolucency (TSR), on the diagnosis of dental caries through a comprehensive analysis of the existing literature [5] .

## Materials and methods

### Protocol and registration

The planning of this systematic review and meta-analysis was conducted following the recommendations of the Cochrane Handbook for Systematic Reviews of Interventions, in accordance with the PRISMA (Preferred Reporting Items for Systematic Reviews and Meta-Analysis) guidelines. The protocol was registered on the PROSPERO (International Prospective Register of Systematic Reviews) platform with the registration number PROSPERO CRD420251083823, available at https://www.crd.york.ac.uk/PROSPERO/view/CRD420251083823 (last accessed: 1 July 2025), before proceeding with the selection of studies to be included and data extraction.

### Eligibility criteria

The study objectives were divided into two primary outcomes: the first aims to assess the prevalence of non-carious TSR, and the second seeks to determine the extent of false-positive diagnoses in which TSR and Mach bands are erroneously interpreted as dental pathologies.

Primary outcome PICO:

P (Population): deciduous and permanent molars subjected to dental radiography;

I (Exposure): presence of non-carious TSR;

C (Comparator): none (prevalence study, no control group);

O (Outcome): prevalence of TSR.

PICO question: In dental radiographs of deciduous and permanent molars, what is the prevalence of non-carious triangular-shaped radiolucencies ?

Secondary outcome PICO:

P (Population): radiographic effects (TSR or Mach-band) assessed by clinicians or students;

I (Intervention/Exposure): diagnostic interpretation of the radiographic optical effects/phenomena, as caries or fracture;

C (Comparator): none (proportion study, no control group);

O (Outcome): proportion of incorrect diagnoses (false positives).

PICO question: In observers evaluating radiographic effects (TSR or Mach-band), what proportion are erroneously diagnosed as carious or fractured lesions?

Inclusion criteria: Original research articles, both clinical and in vitro or ex vivo, that specifically investigate the impact of TSR and/or Mach band effect on intraoral radiographs (bitewing or periapical) are considered eligible. Studies must report at least one of the following diagnostic outcomes: sensitivity, specificity, false positive/false negative rates, interobserver agreement, lesion size estimation or optical effects/phenomena characterization. There are no restrictions on publication date or full text language, as long as an abstract is available in English, and the full text is accessible (scientific article, thesis or conference proceedings). Researches evaluating methods aimed at mitigating these radiographic optical effects/phenomena (beam angle, centering systems, masking techniques or digital post-processing), are also included.

Exclusion criteria: Narrative reviews, systematic reviews and meta-analyses are excluded (the references of which may however be explored to identify primary studies). Single case reports or case series with fewer than five subjects/teeth are not permitted unless they present a detailed quantitative analysis of the radiographic optical effects/phenomena. Studies dedicated exclusively to non-intraoral techniques (panoramic, Cone beam computed tomography, CBCT, optical coherence tomography, OCT) without comparison with intraoral radiography), research on animal models and purely computational simulations without real radiographic data are also excluded.

### Sources of information, research and selection

Literature searches for eligible studies and manuscripts were performed by two reviewers (M.D. and D.S.), who are also authors of this manuscript, utilizing online bibliographic databases [6]. No preliminary exclusion criteria were applied, nor were any automated database filters employed to limit the number of retrieved records. The primary platforms interrogated were the Cochrane Library, PubMed and Scopus. In addition, Grey literature was sought via Google Scholar, ScienceDirect and the DANS archive (Data Station Life Sciences) [7]. To mitigate publication bias, reference lists of prior reviews on Mach bands and TSR were hand-searched. The final update of all identified records was completed on 13 June 2025.

Search terms were chosen to capture the widest possible range of studies addressing TSR and Mach-band effects. In each database, the following terms were used in combination: “triangular-shaped radiolucencies” AND “Mach band”.

- PubMed: triangular-shaped radiolucencies OR Mach band.
- sorted by “Most Recent”.
- Scopus: TITLE-ABS-KEY (“triangular-shaped radiolucencies” OR “Mach band”).
- Cochrane Library: the same keywords were applied to titles and abstracts.

The identified records were imported into EndNote 8, and duplicates were automatically detected and removed using the software’s “Find Duplicates” function. Any remaining duplicates not flagged by the software were subsequently eliminated manually by the reviewers responsible for study selection.

Study eligibility was assessed independently by two reviewers (M.D. and C.Q.). Initially, all potentially relevant records were collated and listed, then logged in two separate tables which were compared for consistency. Title screening was used to identify candidate studies, and final inclusion was determined by full-text review.

Inter-reviewer agreement was evaluated, and any discrepancies were adjudicated by a third reviewer (A.T.).

### Data collection process and data characteristics

During the protocol’s preparatory phase, we determined the specific data elements to compilate our summary tables. In keeping with the study selection and screening process, two reviewers independently extracted these data and then performed cross-checks to minimize inaccuracies. Following reconciliation, a single reviewer collated the verified entries into a unified summary table.

Any discrepancies noted during extraction were logged and addressed through in-depth discussions between the two reviewers to resolve misunderstandings and correct potential errors. Persisting disagreements were escalated to a third reviewer, who adjudicated the contested items and provided a definitive assessment of their accuracy. This procedure ensured that all conflicts were resolved in a systematic, transparent manner, grounded in the available evidence.

From each included article, we extracted the following variables: first author; year of publication; study design; country of origin; number of participants and mean age; number and type of teeth analyzed and their anatomical location; number of observers; number of radiographs and imaging modalities employed; counts of non-carious TSR and their locations; counts of Mach-band effects; and number of false-positive diagnoses [8].

### Risk of bias in individual and across studies, synthesis measures and results, publication bias

A strong focus was hired on evaluating the risk of bias using the Appraisal Tool for Cross-Sectional Studies (AXIS), which is well suited to both retrospective cross-sectional investigations and descriptive cohort studies without control groups, especially those reporting prevalence data. Any study judged to carry a high risk of bias was excluded from the meta-analysis. Two reviewers (M.D. and F.E.) carried out the bias assessment independently, and a third reviewer (M.E.B.) adjudicated any disagreements. The final judgements were then synthesized into a single summary table.

Review results are presented in both tabular and graphical form. Graphical outputs include forest plots alongside measures of heterogeneity (Higgins’ I²), while between-study bias was appraised visually by examining confidence-interval (CI) overlap and quantified via the I² statistic (with values > 50% indicating moderate heterogeneity). In instances of substantial heterogeneity, sensitivity analyses were performed by omitting studies with minimal CI overlap or disproportionately large statistical weight.

For the meta-analysis of non-carious TSR prevalence across all teeth, we utilized OpenMeta [Analyst] version 10 (Centre for Evidence-Based Medicine, Brown University) [9]. Publication bias was assessed using a bespoke Windows application (“Funnel Plot Single Arm et al.”), developed in Python by M.D. under A.M.’s supervision and incorporating the numpy, statsmodels.stats.meta_analysis and scipy.stats libraries [10]; this software is provided as supplementary material. Heterogeneity was further evaluated with Cochran’s Q test. Finally, the overall certainty of evidence was appraised using the GRADE online tool, with findings summarized in a GRADE evidence Table [11].

## Results

### Selection of studies

Searches conducted in SCOPUS, PubMed, and the Cochrane library yielded a total of 640 bibliographic records. After removal of duplicates (*n* = 51), 589 records remained and were screened by title and abstract, resulting in nine potentially eligible articles for full-text assessment. Of these, only three articles met the inclusion criteria for the systematic review and meta-analysis, corresponding to the two prespecified outcomes.

**Fig. 1:**
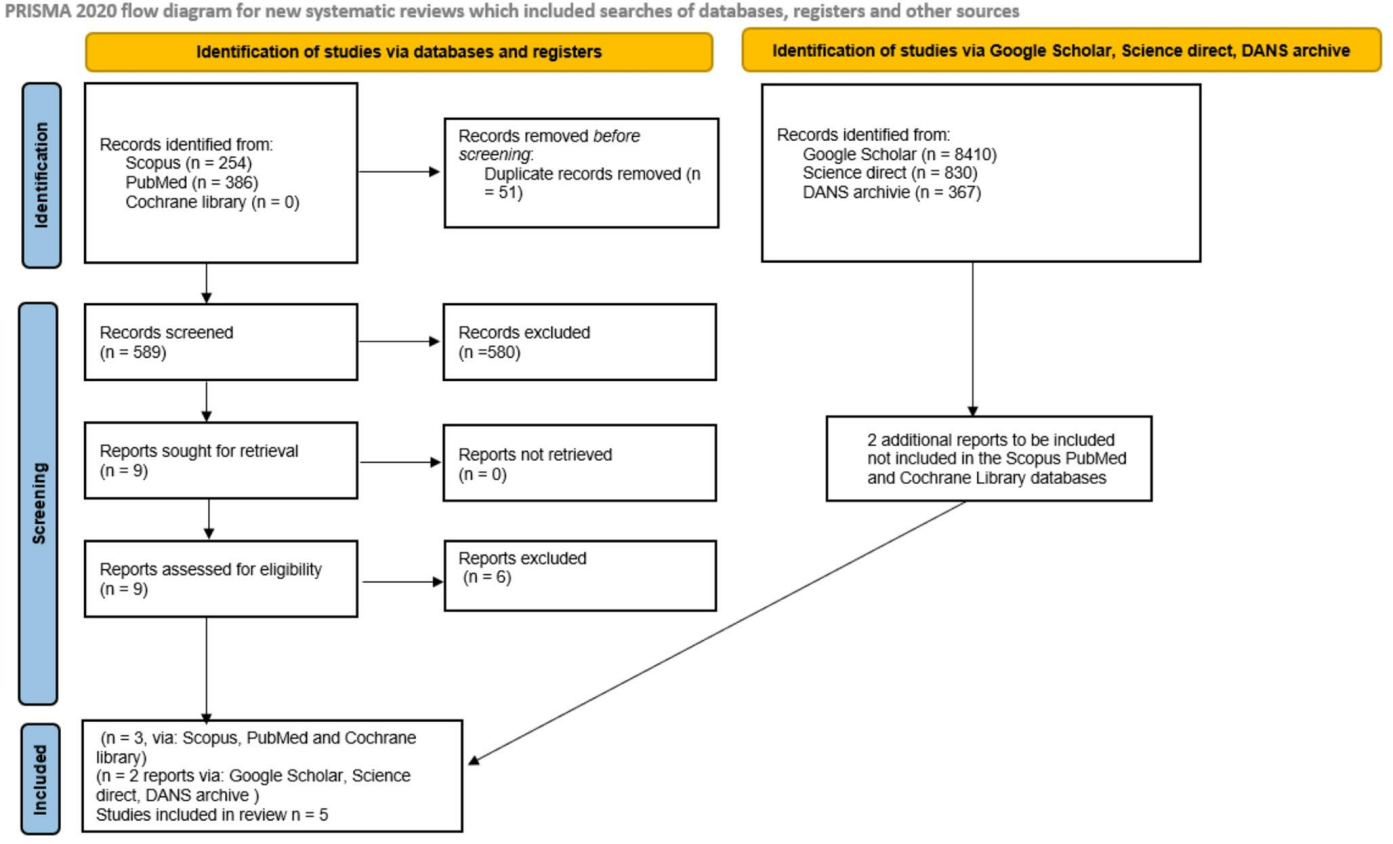
Flow chart of the study selection and inclusion process

In addition, Grey literature searches performed in repositories such as DANS (Data Station Life Sciences) (https://ssh.datastations.nl/), ScienceDirect, Google Scholar, and previous systematic reviews using the keywords “triangular-shaped radiolucencies OR Mach band” identified two further studies suitable for inclusion in the meta-analysis. However, the results of the selection and inclusion process demonstrate that there is an insufficient number of studies (only two) to perform a robust meta-analysis and determine a precise estimate of the prevalence of non-carious TSR in dental radiographs. The complete process of study identification, selection, and inclusion is illustrated in the PRISMA flow diagram shown in Fig. 1.

During the identification phase, nine potentially eligible studies were retrieved via Scopus and PubMed. Of these, six studies were excluded for the following reasons:

- Two studies were reviews that were not explicitly identified as such in the title or abstract (Category B);
- Two studies were case reports that were not explicitly identified as such in the title or abstract (Category A);
- Two studies were in vitro or in vivo investigations which, although they addressed the Mach band effect or TSR, did not report data specifically relating to teeth (Category C).

The excluded studies, together with their respective reasons for exclusion, are summarized in Table 1.

**Table 1:** Excluded studies and reasons for exclusion

Data, autor	country	type of study	reasons for exclusion	
Berry, 1983 [12]	USA	Review	Review	B
Warnock et al., 1985 [13]	USA	Case report	Case report	A
Henning et al., 2004 [14]	Germany	in vitro, in vivo	No data on Mach band or TSR in relation to teeth	C
Kakuma et al., 2008 [15]	Japan	in vitro, in vivo	No data on Mach band or TSR in relation to teeth	C
Melo and Escaffi., 2010 [16]	Chile	Review	Review	B
Laungani et al., 2020 [17]	USA	Case report	Case report	A

Studies excluded after full-text screening with justification based on predefined eligibility criteria. (A): Case report not reported in the abstract or in the title; (B): Review not reported in the abstract or in the title; (C): No data on Mach band or TSR in relation to teeth

1. Berry, 1983 provided a review article (not indicated as such in the abstract or title) that includes clinical illustrations. Berry discusses, in a descriptive and practical manner, the phenomena of “cervical burnout” and the Mach band in dental radiology, proposing diagnostic solutions to distinguish these radiographic optical effects/phenomena from true carious lesions. The author emphasizes the need for clinicians to be aware of these radiographic optical effects/phenomena, to avoid misdiagnosis and recommends combining radiographic and clinical examination, as well as employing simple practical measures such as the masking test, to improve differential diagnosis [12].
2. Warnock et al., 1985 reported a case report, the nature of which was not specified in either the abstract or the title. The article describes a case of squamous odontogenic tumour, a benign epithelial odontogenic neoplasm, which appears radiographically as a well-defined radiolucent area between dental roots [13]. The paper presents the clinical and radiographic features, histological findings, and a conservative surgical approach with follow-up [14].
3. Henning et al., 2004 presented an in vivo and in vitro study on the masking of Mach bands, offering new insights into the limits of visual perception and strategies for isolating the Mach band effect in detection tasks [15].
4. Kakuma et al., 2008 conducted an experimental imaging study, describing the development and validation of an optical coherence tomography (OCT) system (OFDR-OCT, based on frequency-domain reflectometry) with C-band and L-band laser sources for the visualization of dental hard and soft tissues, both in vitro and in vivo [16].
5. Melo and Escaffi, 2010 reported a review article (again, not identified as such in the title or abstract), which is therefore excluded. This paper provides a clear theoretical and practical explanation of the Mach band effect in radiology, emphasizing the importance of recognizing and distinguishing this optical effect for correct image interpretation [17].
6. Laungani et al., 2020 reported a case report (not specified as such in the title or abstract) of a squamous odontogenic tumor, where the lesion was suspected following a radiological finding of a pericoronal radiolucency [18].

### Data characteristics

The manuscripts included in this systematic review, comprise five studies published between 2001 and 2021: two addressing the primary outcome (Khayam et al., 2013 [19]; Kühnisch et al., 2008 [5]) and three pertaining to the secondary outcome (Movahhedian et al. 2017 [20], ; Tabari et al., 2021 [21] ; Nielsen et al., 2001 [22]).

The extracted data are presented in two summary tables (Table 2e Table 3).

For the assessment of TSR prevalence, two observational studies were included. Both studies analyzed bitewing radiographs from large cohorts, comprising individuals with either deciduous or permanent dentition. The data collected covered both children and young adults and included mesial surfaces of various types of molars. In total, 257 patients and 1021 teeth were evaluated.

**Table 2:** Prevalence of Non-Carious Triangular-Shaped radiolucencies (TSR) on maxillary molars: summary of included Studies; prevalence (%) = (Events / Total) × 100, rounded to one decimal place; the data refer to the presence of non-carious TSR on the mesial surfaces of maxillary molars

Author, Reference	Country	Study Design	Sample (Teeth/Surfaces)	Tooth Type	Events (TSR / Total)	Prevalence (%)
Kühnisch et al., 2008 [5]	Germany	Cross-sectional	113 children (11–12 y), 445 surfaces, bitewing	Primary first molar	27 / 78	34.6
				Primary second molar	85 / 141	60.3
				Permanent first molar	56 / 226	24.8
Khayam et al., 2013 [19]	Iran	Retrospective	144 adults (18–24 y), 576 molars, 288 bitewings	Permanent first molar	85 / 288	29.5
				Permanent second molar	17 / 288	5.9

In Kühnisch et al. (2008), the prevalence of TSRs was reported for each surface, rather than each tooth, and the analysis was limited exclusively to the mesial surfaces of maxillary molars (i.e., one surface *per* tooth for each considered molar) [5].

Khayam et al. (2013) reported data for maxillary first and second molars, with events (TSR) occurring in 85 out of 288 first molars and 17 out of 288 s molars. No TSR were reported in mandibular molars or on distal surfaces [19].

**Table 3:** Summary of extracted data on false positive (FP) diagnoses due to radiographic optical effects/phenomena (TSR and Mach Band)

Autor data, reference	Country	Setting	Observers / Evaluations	Optical Effects	Type of False Positive Diagnosis	FP	Total	% FP	Notes
Nielsen 2001[22]	USA	Simulated case	73 participants	Mach band	Fracture	19	73	26.0	Mach band simulated as fracture; single answer per observer
Movahhedian et al. 2016 [20]	Iran	In vitro	94 observers × 3 teeth	TSR	Caries	36	282	12.8	Each observer evaluated multiple TSRs; clustered data
Tabari et al. 2021 [21]	Iran	In vivo	109 teeth (paediatric patients)	TSR	Caries	5	109	4.6	Radiographic vs. clinical gold standard; 2 × 2 contingency

**Fig. 2:**
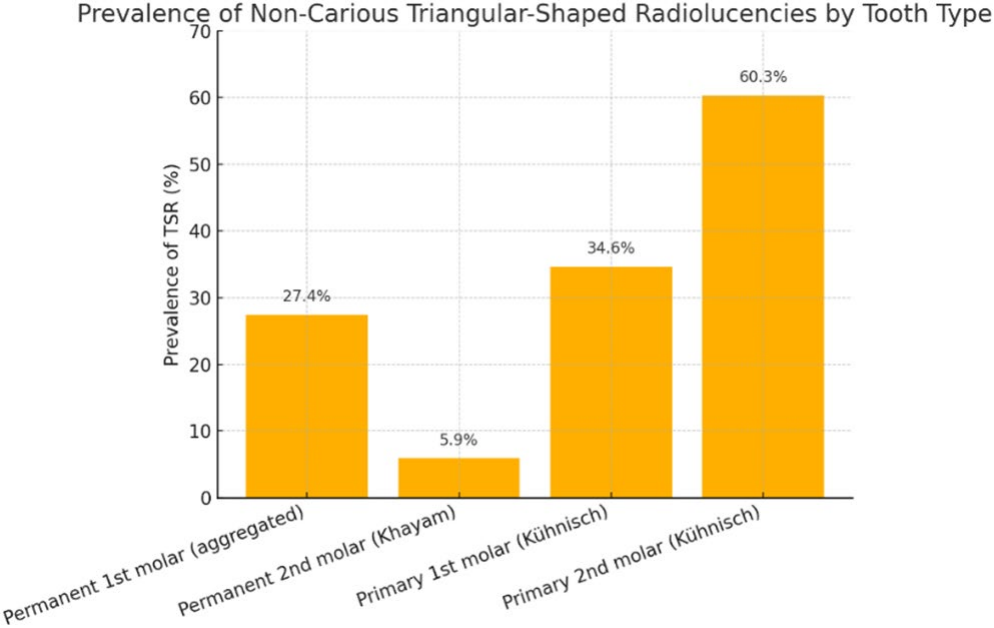
Prevalence of Non-carious TSR by Tooth Type: This bar graph shows the prevalence (%) of non-carious TSR) detected in bitewing radiographs, grouped by tooth type and study source. Data are shown for first and second permanent molars (from Khayam et al., 2013 and Kühnisch et al., 2008), as well as first and second deciduous molars (Kühnisch et al., 2008). TSRs are significantly more prevalent in second deciduous molars (60.3%) than in permanent molars, highlighting

**Fig. 3:**
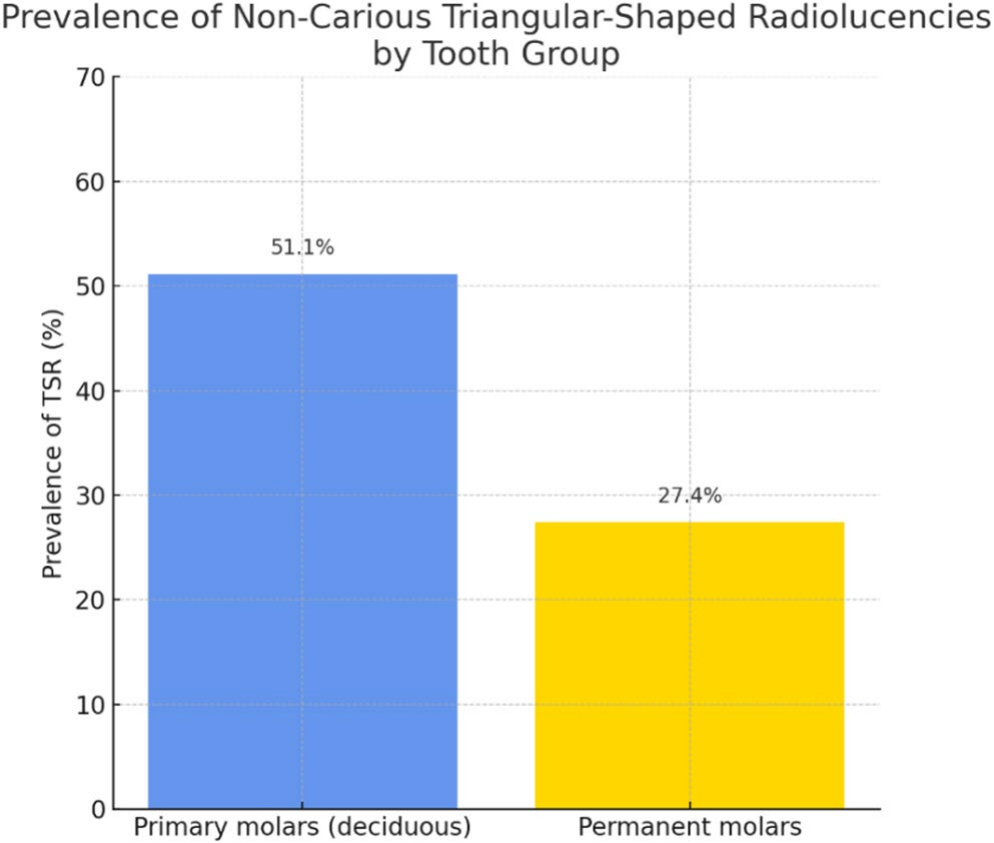
Prevalence of Non-Carious TSR by Tooth Group. This bar chart compares the prevalence (%) of non-carious TSR between primary (deciduous) and permanent molars, as determined from pooled data in the included studies. The results highlight a substantially higher prevalence of TSR in primary molars (51.1%) compared to permanent molars (27.4%)

Furthermore, two graphical representations (Figs. 2 and 3), clearly illustrate that the prevalence of TSR is markedly higher in deciduous molars compared to permanent molars, both when considering individual tooth types and when data are pooled by dental group. Specifically, TSRs are most frequent in second primary molars (exceeding 60%), and when all primary molars are considered together, the prevalence approaches 51%, compared to 27% observed in permanent molars.

This evidence suggests that the risk of radiographic false positives due to TSR, is significantly greater in children with primary dentition, particularly when examining second maxillary primary/ deciduous molars.

Such variability in prevalence, as highlighted by the extracted data, forms the basis for the subsequent quantitative meta-analysis and underscores the importance of a differentiated diagnostic approach when evaluating TSR in primary versus permanent dentition.

an increased risk of radiographic optical effects/phenomena in the pediatric population.

For the secondary outcome, were selected studies in which data were extracted regarding the frequency were radiographic optical effects/phenomena, such as non-carious triangular-shaped radiolucencies or the Mach band phenomenon, were erroneously interpreted by clinical observers or students, as dental pathologies (caries or fractures).

**Fig. 4:**
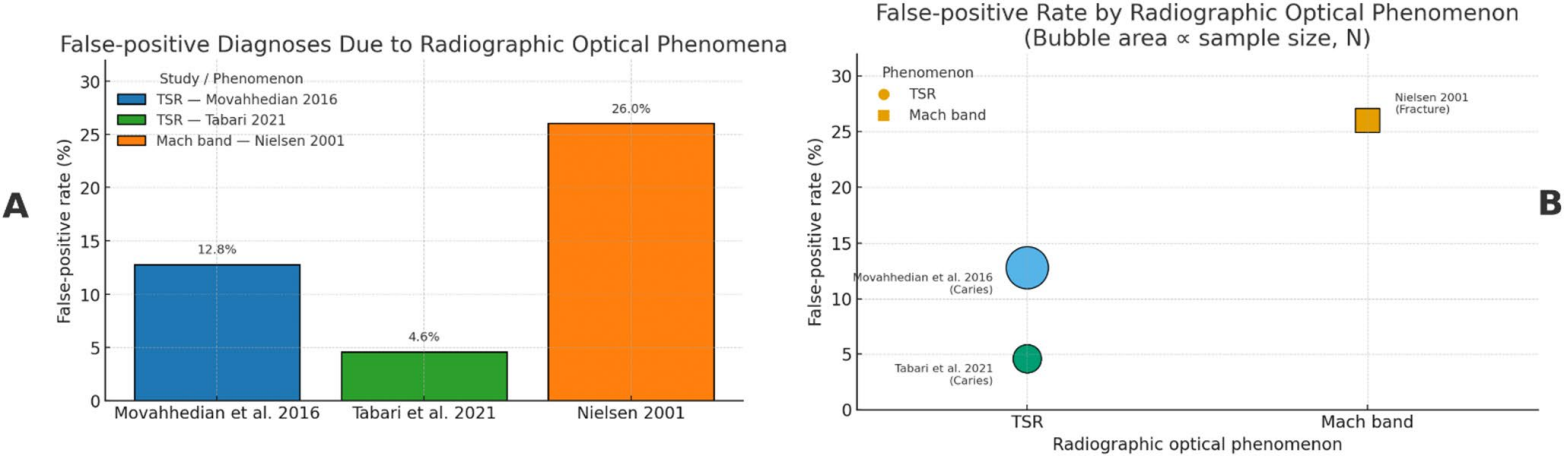
A: shows the percentage of false positives due to radiographic optical effects in the three included studies. This bar chart illustrates the proportion of false positive diagnoses attributed to radiographic optical effects—specifically TSRs and Mach band phenomena—in three different studies. The rates of false positives, representing incorrect interpretations of optical effects as carious or fractured lesions, range from 4.6% (Tabari et al., 2021) to 26.0% (Nielsen, 2001). The findings demonstrate considerable variability across studies, reflecting differences in radiographic optical effects/phenomena type, observer experience, and study design. B: relates the type of radiographic optical effects (TSR or Mach band) to the percentage of false positives. The size of the bubble represents the sample size for each study; the color differentiates studies, TSR (buble) and Mach band (square). The labels report author/year and the prevalent incorrect diagnosis (caries or fracture)

The collected data encompassed both in vitro and in vivo settings, as well as simulated scenarios, and included assessments involving paediatric samples, experienced dental practitioners, and students. For each study, information was extracted on the number of incorrect diagnoses (false positives), the total number of observations, the type of effects evaluated, and the nature of the diagnostic error (caries or fracture). The proportion of false positives reported across the different studies, ranged from approximately 5% to 26%, highlighting a certain degree of heterogeneity related to the type of a radiographic optical effects/phenomena, as well the experience level of the observer, and the experimental conditions (Fig. 4).

Taken together, these data enable an estimation of the impact of radiographic optical effects on the frequency of diagnostic errors in dental practice, thus providing a quantitative basis for the subsequent meta-analysis.

### Meta-analysis

The meta-analyses were performed using OpenMeta [Analyst] version 10, which generated forest plots to visualize the aggregated results. These tools were used to synthesize and analyze the pooled data from the included studies, providing a comprehensive overview of the evaluated outcomes.

**Fig. 5:**
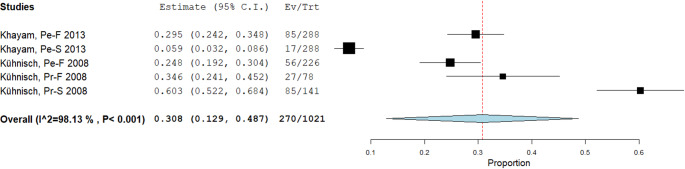
Binary Random-Effects Model, Metric, Proportion. Estimate 0.308, Lower bound 0.129, Upper bound 0.487, Std. error 0.091, p-Value < 0.001, tau^0.040, Q(df = 4) 213.888, Het. p-Value < 0.001, I^2 98.13. The apparent discrepancy reflects the random-effects (DerSimonian–Laird) pooled prevalence = 0.308 (95% CI 0.129–0.487) across five datasets (Khayam 2013 × 2; Kühnisch 2008 × 3), whereas 26.44% is the raw, unweighted proportion (270/1,021). With marked heterogeneity (I² ≈ 98%), the inverse-variance–weighted random-effects estimate, is not expected to equal the raw proportion

The first meta-analysis focused on the prevalence of non-carious TSR in maxillary molars. This analysis included two studies (Khayam et al., 2013 [19]; Kühnisch et al., 2008 [5]), encompassing five datasets. A random-effects model, as described by DerSimonian and Laird, was applied to calculate the cumulative proportion of TSR events in relation to the total number of maxillary molars examined. The final result was 270 TSRs out of 1,025 maxillary molars, corresponding to a prevalence of 26.44% (Fig. 5).

A subgroup analysis was also performed according to molar type (permanent or primary):

- Permanent subgroup (A): Based on two studies and three datasets;
- Primary subgroup (B): Based on two studies and two datasets.

**Fig. 6:**
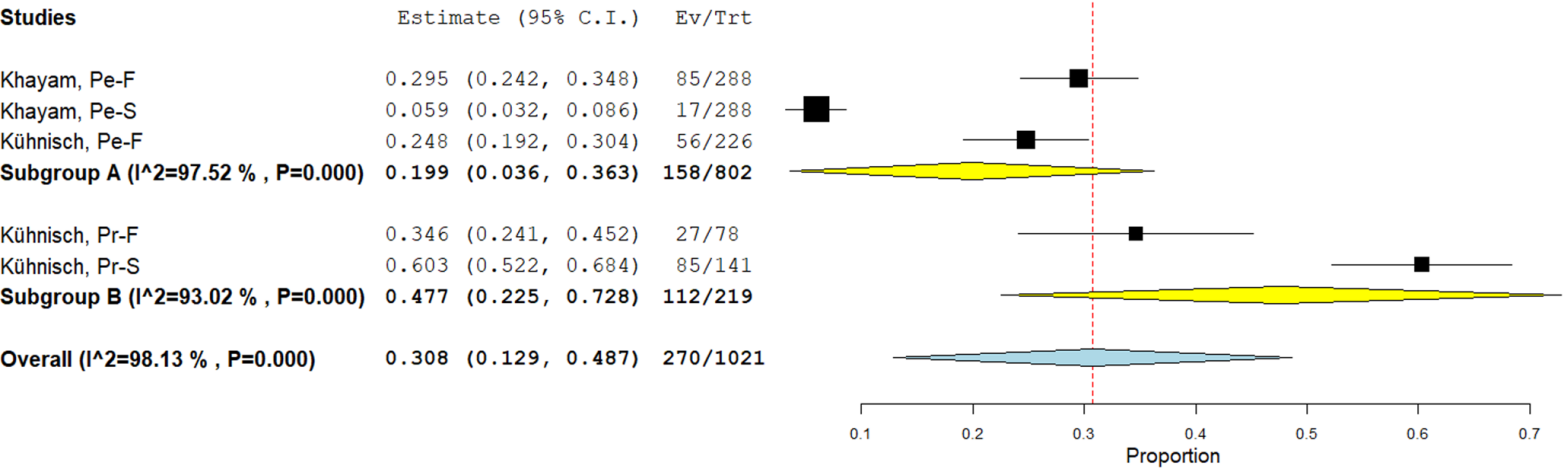
Subgroup analysis — Binary random-effects model (DerSimonian–Laird), metric: proportion. Forest plot showing the estimated prevalence of non-carious TSR for each dataset, pooled by subgroup (A: permanent molars; B: primary molars) and overall. Horizontal lines indicate 95% confidence intervals; symbol width reflects study weight; the vertical dashed line marks the overall pooled estimate. Subgroup estimates: permanent = 0.199 (95% CI: 0.036–0.363; Q[df = 2] = 80.692; *p* < 0.001; I² = 97.5%), primary = 0.477 (95% CI: 0.225–0.728; Q[df = 1] = 14.324; *p* < 0.001; I² = 92.1%). Overall pooled prevalence = 0.308 (95% CI: 0.129–0.487), with substantial heterogeneity (I² > 90%)

As shown in Fig. 6, the estimated value for the permanent subgroup was 158 TSR on 802 molars (0.199), whereas for the primary subgroup it was 112 TSR on 223 molars (0.469). All data relating to the subgroups are detailed in Table 4.

**Table 4:** Pooled prevalence and heterogeneity statistics for TSR by molar type

Subgroup	Data set (*n*)	Estimate	Lower bound	Upper bound	Std. error	*p*-Value	z-Value	Q (df)	Het. *p*-Value	I² (%)
permanent molar	3	0.199	0.036	0.363	0.083	0.017	2.395	80.692 (2)	< 0.001	97.52
Primary molar	2	0.477	0.225	0.728	0.128	< 0.001	3.716	14.324 (1)	< 0.001	92.06
Overall	5	0.308	0.129	0.487	0.091	< 0.001	3.371	–	–	–

This table summarizes the pooled prevalence estimates of non-carious TSR for permanent and primary molars, based on a random-effects meta-analysis of the included datasets. For each subgroup and for the overall analysis, the table reports the number of datasets, the estimated prevalence (proportion), 95% confidence intervals (lower and upper bounds), standard error, p-value, z-value, Q statistic (with degrees of freedom-df), the p-value for heterogeneity, and the I² index indicating the degree of between-study heterogeneity. High I² values (greater than 90%) indicate substantial heterogeneity across studies

The second meta-analysis addressed the secondary outcome, with the following PICO question: In observers evaluating radiographic optical effects/phenomena (TSR or Mach-band), what proportion are erroneously diagnosed as carious or fractured lesions? Specifically, this analysis focused on the percentage of false positives attributable to radiographic optical effects/phenomena.

**Fig. 7:**
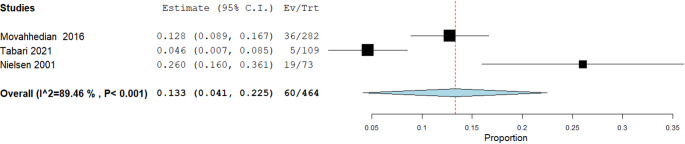
False-positive diagnoses attributable to radiographic optical effects — Binary random-effects model (DerSimonian–Laird), metric: proportion. Forest plot showing the pooled proportion of false-positive diagnoses where TSR or Mach-band phenomena were misinterpreted as pathology. Pooled estimate = 0.133 (95% CI: 0.041–0.225; SE = 0.047; *p* = 0.004); between-study variance τ² = 0.006; heterogeneity Q(df = 2) = 18.971, *p* < 0.001; I² = 89.46%. Inverse-variance weights: Movahhedian 36.683%, Tabari 36.642%, Nielsen 26.675%. The vertical dashed line marks the pooled estimate; horizontal lines indicate study 95% CIs. Substantial heterogeneity likely reflects differences in setting (in vivo / in vitro / simulation), observer experience, and optical-effect type (TSR vs. Mach band)

A random-effects model, as described by DerSimonian and Laird, was applied, given the heterogeneity of the included studies—to calculate the cumulative proportion of false positive events relative to the total number of observations. The final result was 60 false positives out of 464 observations, corresponding to a prevalence of 12.93% (Fig. 7).

### Publication bias

For each study, data were transformed using the logit function, with continuity correction applied to stabilise variance in the presence of rare events: logit(p) = ln[(events + 0.5)/(total + 1 − events − 0.5)].

The pooled effect estimate was calculated using a fixed-effects model with inverse variance weighting. When fewer than five to seven studies are included, the estimate of between-study variance (τ²) in random-effects models is unstable and may introduce more unuseful data than information. The fixed-effects model avoids overestimating uncertainty when τ² cannot be reliably estimated, especially given that the analyzed subgroups were methodologically homogeneous, the number of studies was limited, and the objective of the funnel plot was purely diagnostic (identification of asymmetries). Thus, adoption of a fixed-effects model provides a stable graphical reference, without introducing the additional, and largely uninformative uncertainty associated with τ² when only a small number of studies are available.

**Fig. 8:**
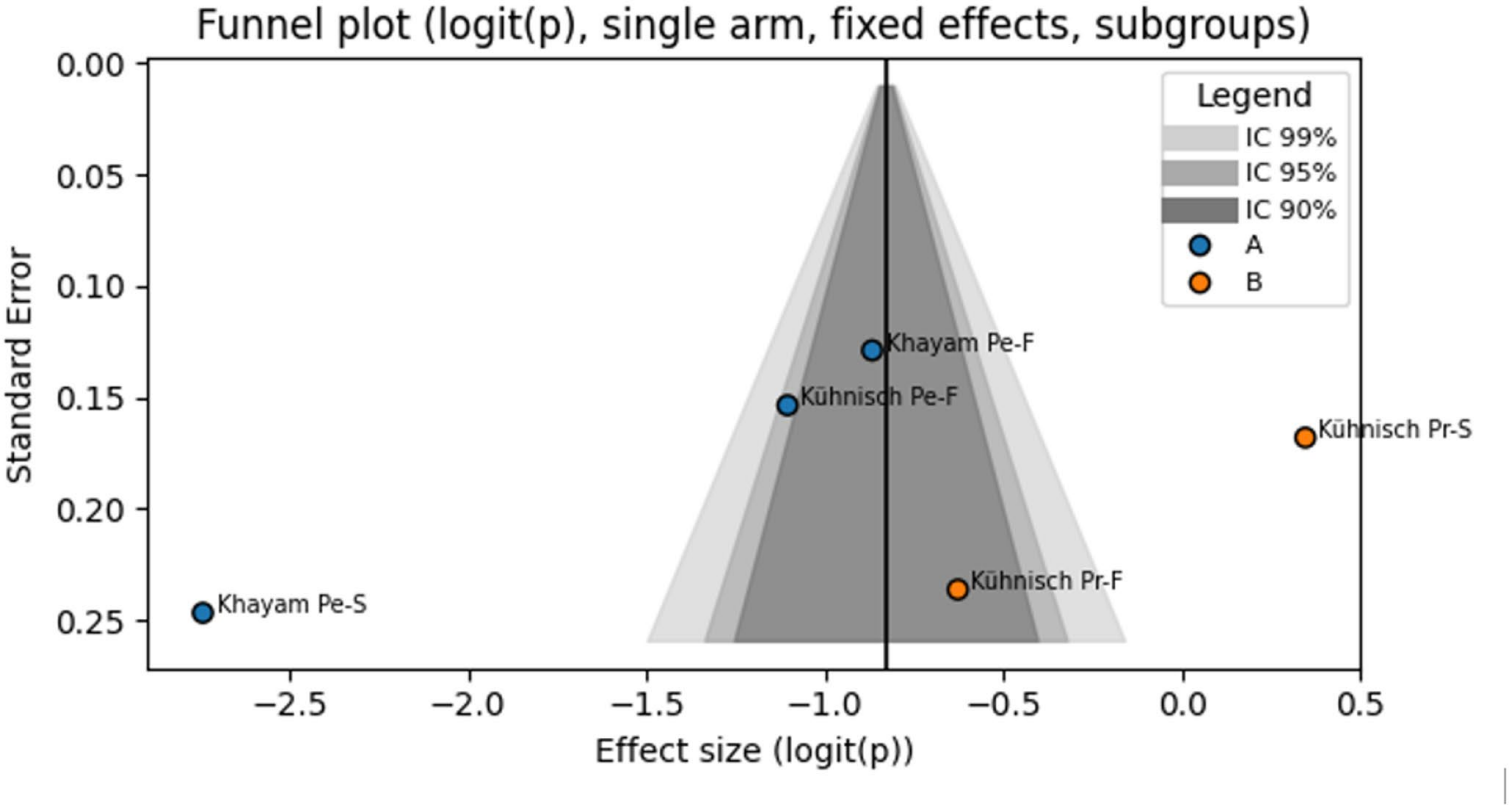
Funnel Plot of included studies (Logit-Transformed Proportions, Fixed Effects Model), Assessment of publication bias based on logit-transformed proportions with continuity correction. Shaded areas represent 99%, 95%, and 90% confidence intervals; A : subgroup permanent, B subgroup primary, Pe-F: Permanent first molar, Pe-S: Permanent second molar, Pr-F: Primary first molar, Pr-S: Primary second molar, Group A/B: meta-anlysis soubgropus, logit(p): Logit transformation of the proportion, SE: Standard Error

Funnel plot symmetry was assessed both visually and by means of Egger’s test, which estimates the regression intercept between the standardized effect and study precision (1/SE). The funnel plot was enhanced with confidence curves at the 99%, 95%, and 90% levels in a greyscale gradient, together with subgroup-specific color coding to facilitate the identification of visual asymmetries potentially related to study characteristics. All graphs and summary tables were exported directly from the application interface (Fig. 8) (Table 5).

**Table 5:** Egger’s test for funnel plot asymmetry egger’s regression test was conducted to formally assess funnel plot asymmetry

Egger’s Test Results	Value
Intercept (β₀)	-6.9069
Std Error	10.7911
t-value	-0.6401
DF	3
p-value	0.5677
95% CI	[-28.0574; 14.2435]

The test yielded an intercept (β₀) of − 6.91 (standard error: 10.79), with a t-value of − 0.64 (degrees of freedom: 3) and a p-value of 0.5677. The 95% confidence interval for the intercept ranged from − 28.06 to 14.24. These results do not provide evidence of significant asymmetry, suggesting the absence of a strong publication bias; however, the limited number of included studies reduces the power of this assessment

The results of Egger’s test (intercept = − 6.91, *p* = 0.5677; 95% CI: − 28.06 to 14.24), did not indicate evidence of publication bias among the included studies. Similarly, visual inspection of the funnel plot revealed no clear asymmetry, suggesting that small-study effects are unlikely to have significantly influenced the pooled estimates. However, it is important to emphasize that both the statistical test and the funnel plot have limited power when the number of included studies is small, as is the case in the present review. Consequently, the absence of detected bias should be interpreted with caution.

### Risk of bias

The risk of bias assessment for the primary outcome was conducted using the AXIS tool, which is specifically designed for cross-sectional studies and is particularly suitable for prevalence analyses. The results are summarized in Table 6, where two reviewers independently evaluated 20 items, assigning one of the following judgements to each study: Yes, No, or Do not know [23].

**Table 6:** AXIS tool assessment of methodological quality in included studies on oral manifestations of LCH: synthesis of responses provided by two independent reviewers to the 20-item AXIS checklist. Answers are reported as ‘Yes’, ‘No’, or ‘Do not know’

AXIS Item	Khayam et al., 2013	Kühnisch et al., 2008
1. Were the aims/objectives of the study clear?	Yes	Yes
2. Was the study design appropriate for the stated aim(s)?	Yes	Yes
3. Was the sample size justified?	No (not justified/calculated)	No (not justified/calculated)
4. Was the target/reference population clearly defined?	Yes (adults, 18–24 years)	Yes (children, 11–12 years)
5. Was the sample frame taken from an appropriate population base?	Yes	Yes
6. Was the selection process likely to select a representative sample?	Yes (consecutive sampling)	Yes (longitudinal study cohort)
7. Were measures undertaken to address and categorise non-responders?	N/A (not applicable, retrospective analysis)	N/A (not applicable)
8. Were the risk factor and outcome variables appropriate to the aims?	Yes (TSR, molar surfaces)	Yes (TSR, molar surfaces)
9. Were the risk factor and outcome variables measured correctly using valid instruments?	Yes (trained examiner, test-retest)	Yes (calibrated observers)
10. Was it clear what was used to determine statistical significance and/or precision estimates?	Yes (chi-square, p-value)	Yes (clear description, p-value)
11. Were the methods (including statistical methods) sufficiently described?	Yes	Yes
12. Were the basic data adequately described?	Yes (detailed tables)	Yes (detailed tables)
13. Does the response rate raise concerns about non-response bias?	No (all eligible radiographs included)	No (nearly complete participation)
14. If appropriate, was information about non-responders described?	N/A (not foreseen)	N/A (not foreseen)
15. Were the results internally consistent?	Yes	Yes
16. Were the results for the analyses described in the methods, presented?	Yes	Yes
17. Were the authors’ discussions and conclusions justified by the results?	Yes	Yes
18. Were the limitations of the study discussed?	Yes (limitations acknowledged)	Yes (limitations mentioned)
19. Were there any funding sources or conflicts of interest that may affect interpretation?	Yes (none declared)	Yes (not specifically mentioned, but no evidence of conflicts)
20. Was ethical approval or consent of participants attained?	Yes (consent described)	Yes (ethical approval reported)

Both Khayam et al. (2013) [19] and Kühnisch et al. (2008) [5], clearly stated their research objectives and adopted appropriate study designs for assessing the prevalence of TSR in molar radiographs. The target and reference populations were well-defined, and the selection of participants was intended to provide representative samples within their respective contexts (young adults for Khayam [19] and school-aged children for Kühnisch [5]).

Neither study justified or calculated the sample size, which is a common limitation in prevalence research and may affect the precision of the reported estimates. Nevertheless, all eligible radiographs or participants were included, thereby minimizing the risk of selection bias. Given the retrospective and cross-sectional nature of these investigations, issues related to non-responders were not relevant. Both studies relied on qualified and calibrated examiners, and their measurement methods as well as statistical analyses were described with sufficient clarity to ensure replicability.

The results were presented transparently, with detailed data tables, and were found to be internally consistent. The authors provided interpretations and conclusions that were well justified by their findings and explicitly discussed the limitations of their work. No relevant conflicts of interest or sources of funding were identified, and both studies documented appropriate ethical procedures.

In summary, although the lack of a formal justification for sample size represents a minor methodological limitation, both studies demonstrated an overall low risk of bias and were considered to provide reliable data for estimating TSR prevalence in molars.

**Fig. 9:**
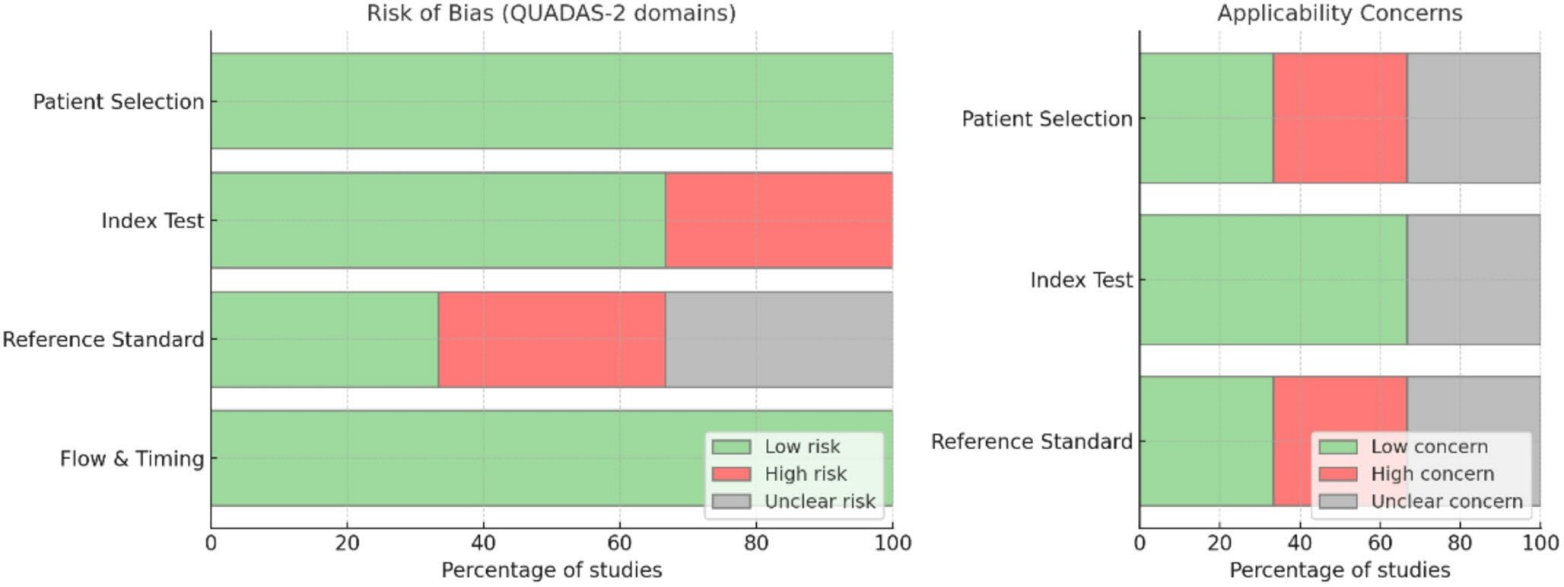
Graphical summary of risk of bias and applicability concerns according to QUADAS-2 domains in the three included studies. On the left: Percentage of studies with risk of bias (green = low, red = high, grey = unclear) for each domain. On the right: Percentage of studies with applicability concerns (green = low, red = high, grey = unclear) for each domain

The risk of bias for the secondary outcome (i.e. for studies evaluating the frequency of false positive diagnoses attributable to radiographic optical effects/phenomena such as TSR and the Mach band), was assessed using the QUADAS-2 tool [24].(Table 7; Fig. 9). In the selected studies, the domains of patient selection and flow and timing were generally considered to be at low risk, as participant recruitment and data collection were adequately described.

**Table 7:** Quadas 2. Low risk / Low applicability concern: the study Meets the methodological criteria for the respective domain, and the results are likely to be reliable and generalizable to the target population

Study	Patient Selection Risk of Bias	Patient Selection Applicability	Index Test Risk of Bias	Index Test Applicability	Reference Standard Risk of Bias	Reference Standard Applicability	Flow & Timing Risk of Bias
Tabari et al.,= 2021	Low risk	Low	Low risk	Low	Low risk	Low	Low risk
Movahhedian et al., 2016	Low risk	Unclear	Low risk	Low	High risk	Unclear	Low risk
Nielsen, 2001	Low risk	High	High risk	Unclear	Unclear	High	Low risk

High risk / High applicability concern: The study has significant methodological shortcomings in the respective domain, or its context substantially limits the generalizability of the findings. Unclear risk / Moderate applicability concern: The information provided is insufficient to make a clear judgement, or the domain is only partially addressed; the results should therefore be interpreted with caution

For Tabari et al., 2021, both the index test (radiographic assessment), and the reference standard (clinical examination), were clearly defined and appropriately applied, resulting in an overall low risk of bias and high applicability to the paediatric clinical setting [21].

Movahhedian et al., 2016 [20], performed an in vitro study using extracted teeth and histological assessment as the reference standard. While this ensured high internal validity, the generalizability to real-world clinical settings is limited, and the risk of bias and concerns regarding the reference standard and its applicability were judged as moderate to high.

Nielsen, 2001, utilized simulated scenarios with dental students and professionals, without a true clinical or pathological gold standard, and focused on interpretive bias [22]. The absence of a reference standard and the artificial nature of the simulation led to a higher risk of bias in the index test and reference standard domains, with limited applicability to clinical decision-making.

Overall, the included studies exhibited variable methodological quality and clinical relevance, with the main limitations arising from the use of simulated or in vitro models, rather than real patient data and reference standards based on clinical or histological confirmation.

### Assessment of quality of evidence (GRADE)

The quality of evidence for both the primary outcome (prevalence of non-carious TSR) and the secondary outcome (proportion of false positive diagnoses due to radiographic opticale effects/phenomena) was assessed using the GRADE approach. As all included studies were observational, the initial level of evidence was considered low.

For the primary outcome, the risk of bias was generally low; however, the quality of evidence was downgraded due to the high inconsistency observed (I² > 90%), and the limited number of available studies. For the secondary outcome, risk of bias and indirectness (for example, in vitro or simulated studies and non-clinical settings) led to a further downgrading of the evidence. Imprecision arising from wide confidence intervals and small sample sizes, as well as the potential for publication bias (although not statistically demonstrated), were also considered.

As a result, the overall quality of evidence for both outcomes was judged to be low to very low. These findings highlight the need for caution in interpreting the pooled estimates and reinforce the importance of further well-designed studies to strengthen the evidence base (Table 8).

**Table 8:** Assessment of quality of evidence (GRADE)

Outcome	Study Design	Risk of Bias	Inconsistency	Indirectness	Imprecision	Publication Bias	Overall Certainty
1 Outcome	Observational	Low	Serious	No	Serious	Possible	Low
2 Outcome	Observational	Moderate-High	Serious	Serious	Serious	Possible	Very Low

## Discussion

This systematic review and meta-analysis provides a quantitative synthesis of the impact of radiographic optical effects, particularly the Mach band effect and non-carious TSR, on the accuracy of dental caries diagnosis. Following a thorough selection process, five studies were included, two for the primary outcome and three for the secondary outcome. The analysis encompassed approximately 1,025 teeth, including both primary and permanent first and second maxillary molars. Radiographic optical effects/phenomena mainly categorized as Mach bands, TSR, and, to a lesser extent, cervical burnout were present in about 26% of maxillary molars (first and second). Notably, TSR were most frequently observed on the mesial surface of maxillary molars.

It is important to highlight that around 13% of these radiographic optical effects/phenomena, were falsely interpreted as carious lesions. Such misinterpretations may lead to unnecessary conservative, endodontic, or even extraction treatments if mistaken for true fractures [25]. In one example, dental practitioners overestimated the presence of carious lesions in over two-third of patients and often treatment planned enamel lesions using invasive therapeutic procedures [26]. These misdiagnoses, could partly be due to erroneous interpretation of radiographic optical effects/phenomena. The occurrence of these optical effects/phenomena in primary molars also raises concerns in paediatric dentistry, where diagnostic overestimation can result in unwarranted treatment. Therefore, the ability to identify and recognize these radiographic optical effects/phenomena is a diagnostic priority, as radiographic examination should support, rather than confound, the clinical diagnosis, reinforcing the central role of thorough intraoral examination.

The most common techniques for identifying and mitigating radiographic optical effects/phenomena that may generate optical effects include the use of digital software, as described by Qaramaleki et al. [27]. Such software reduces the optical illusion caused by the Mach band effect by enhancing the clarity of radiolucent areas in radiographs The software analyses the context of the radiolucent area to diagnose secondary caries in restored teeth and distinguish between true caries and optical illusions. If varying grey levels are detected within a radiolucent area, caries may be present; if not, the finding is more likely attributable to the Mach band effect. Even manual adjustment of contrast can help mask the Mach band effect.

Another widely used technique is the masking test, in which an opaque card is placed over the enamel margin. If the radiolucent area disappears upon covering the enamel, it is likely a Mach band; if it remains visible, it indicates true demineralisation. Mach bands may appear as either black bands (“negative Mach bands”) or white bands (“positive Mach bands”), depending on the geometric relationships and density differences between adjacent structures [17]. A positive Mach band presents as a narrow, lighter band along the margin where a denser (radiopaque) area meets a less dense (radiolucent) region; this accentuates the contrast at the interface, making the boundary more pronounced. Furthermore, a negative Mach band is seen as a narrow, darker band at the border between a radiolucent and a radiopaque area, appearing as a strip even darker than the surrounding tissue. Both positive and negative Mach bands are perceptual illusions created by the visual system, which exaggerate apparent contrast between areas of different density and may lead to diagnostic misinterpretations on radiographic assessment.

Henning et al. (2004) [15], demonstrated experimentally that Mach bands are not only contrast illusions but may also have clinical implications, as they can obscure the detection of small anomalies or objects in the affected area. Thus, Mach bands can act as subtle masks that raise the detection threshold for minor features in their vicinity, owing to the non-linear responses and channel properties of the visual system. In dentistry, this may account for both false positives (radiographic optical effects/phenomena mistaken for carious lesions) and false negatives (true carious lesions masked by the Mach band).

TSR are considered an optical effect, attributed to the superimposition of the Carabelli cusp and the unique anatomy of maxillary molars, a finding later confirmed by Khayam et al. [19]. Their first observation is relatively recent, with Kühnisch et al. (2008) [5] being the first to describe them on the mesial surfaces of both permanent and primary molars. TSRs are also highly frequent in primary first and second molars, being present in around 50% of bitewing radiographs This phenomenon is commonly encountered in paediatric dentistry.

### Limitations of the review

Despite the rigorous methodology applied in conducting and reporting this review, several limitations should be acknowledged, which collectively reduce the certainty and generalizability of the findings. The overall quality of the evidence, as assessed using GRADE, varies from low to very low for both primary and secondary outcomes. This downgrading was mainly due to the observational nature of the included studies, high heterogeneity of the results (I² > 90%), and limited precision caused by the small number of studies and wide confidence intervals. The pronounced inconsistency, particularly in prevalence estimates, further undermines the reliability of pooled effect measures.

Additionally, the risk of bias assessment (AXIS and QUADAS-2) [24] highlighted a number of methodological issues. While the risk of bias was generally low for most domains in clinical studies, significant concerns were identified regarding the reference standard and applicability in some studies. In fact, several included studies did not employ representative patient samples but instead used extracted teeth, laboratory conditions, or dental students as observers, limiting the generalizability of the findings, in particularly with regard to differences between paediatric and adult patients, or between primary and permanent teeth.

Publication bias remains a potential concern; although statistical tests (e.g., Egger’s test) and visual inspection of funnel plots did not confirm significant bias, the limited number of available studies and the predominance of positive findings do not rule out selective publication.

Finally, the systematic review was further limited by the small number and quality of eligible studies. Most available data were derived from cross-sectional or retrospective studies, with a lack of prospective, multicentre, or interventional research. The scarcity of high-quality evidence, the absence of standardized diagnostic criteria for TSR and Mach band effects, and reliance on radiographic rather than clinical or histological confirmation, all further weaken the strength of the conclusions.

These limitations underscore the need for caution in interpreting the findings of this meta-analysis. Additional high-quality, prospective research with robust methodology, standardized definitions, and clinically relevant reference standards, is essential to strengthen the evidence base regarding the diagnostic impact of radiographic optical effects/phenomena in dental practice.

Future research should prioritizes the conduct of well-designed prospective clinical studies to clarify the prevalence and diagnostic impact of radiographic optical effects/phenomena such as TSR and the Mach band effect, as well as the impact of cervical burnout. There is also a pressing need for studies focused on observer training and education, aimed at improving the ability of dentists and students to distinguish radiographic optical effects/phenomena, from true pathological lesions. The development and implementation of artificial intelligence-assisted diagnostic tools or digital post-processing techniques may also prove promising in reducing diagnostic errors associated with radiographic optical effects/phenomena. Overall, such efforts will be crucial for strengthening the evidence base and informing clinical guidelines aimed at minimizing the diagnostic pitfalls of radiographic optical effects/phenomena in dental practice.

## Conclusions

In conclusion, the present systematic review, highlights that radiographic optical effects/phenomena particularly TSR and the Mach band effect, have an overall prevalence of approximately 26% in bitewing radiographs, with TSR most commonly located on the mesial surfaces of upper first molars. According to the meta-analytic data, these radiographic optical effects/phenomena can significantly compromise diagnostic accuracy, as the proportion of false positives radiographic optical effects/phenomena mistaken for carious lesions or dental fractures, was found to be around 13%. Given the low certainty of the current evidence, dental clinicians should exercise caution and supplement radiographic findings with thorough clinical assessment. High-quality research is urgently needed to refine diagnostic approaches and ensure optimal patient care.

## Supplementary Information

Below is the link to the electronic supplementary material.

Supplementary Material 1

## Data Availability

No new data were created. Declarations.
